# Suggestions by the Board of Control

**Published:** 1920-05-15

**Authors:** 


### RATIONING ACCOMMODATION.

### Suggestions by the Board of Control.

During the war there was a progressive falling-off in the number of patients in county and borough asylums in England and Wales, and at present there are many thousands of vacancies in these institutions.

The Board of Control, which is anxious to provide additional accommodation for mental defectives, has come to the conclusion that it might be possible with advantage to utilise—say, for some seven or eight years—some of this vacant accommodation by devoting a few asylums exclusively to the accommodation of mental defectives requiring institutional care.

A War-time Parallel.

Communicating with the authorities concerned, the Board of Control suggests that this might be done by establishing a scheme, less extensive, but somewhat on the lines of the scheme whereby a number of asylums have during the war functioned as war hospitals.

It is suggested that a few of the smaller asylums, situate in as widely different localities as possible, should be cleared of their insane patients and then certified as institutions under the Mental Deficiency Act, each of which would be managed by the Mental Deficiency Committee of the local authority to whom it belongs.

It is also suggested that the patients from each vacated asylum should be boarded out under contract at the out-county rate in neighbouring asylums, and the Mental Deficiency Committees of these local authorities whose Visiting Committees are willing that their institutions should then become receiving asylums, should, under agreement, have the primary right to contract with the certified institution (that is, the vacated asylum) for the reception therein of their mental defectives.

The Board, which has reason to think that there are some authorities who would be willing for a time to give up their asylums so as to enable them to be used as mental deficiency institutions, points out that some of the advantages of the scheme proposed would be :—

Some Possible Advantages.

(1) The receiving asylums, by filling up a number of their vacant beds, would be able to maintain their own patients at a somewhat lower cost than they otherwise could, and would also receive an additional amount above their maintenance rate by way of rent for the accommodation so occupied.

(2) The smaller asylums on becoming certified institutions would avoid the extravagant cost for maintenance which would necessarily result from their continuing to provide in their own institution for the comparatively small number of their insane patients, and should obtain profit from the mental defectives they would accommodate in their place.

(3) The necessity for an immediate very large outlay in building operations for the erection of certified institutions would to a considerable extent be obviated.

(4) The arrangement would give the various Mental Deficiency Committees further time in which to consider their position, and to mature their schemes for the provision of permanent accommodation for their mental defectives.

